# Dysphagia, health-related quality of life, and return to work after occipitocervical fixation

**DOI:** 10.1007/s00701-024-05991-6

**Published:** 2024-02-20

**Authors:** Aman Singh, Ann-Christin von Vogelsang, Charles Tatter, Victor Gabriel El-Hajj, Alexander Fletcher-Sandersjöö, Paulina Cewe, Gunnar Nilsson, Simon Blixt, Paul Gerdhem, Erik Edström, Adrian Elmi-Terander

**Affiliations:** 1 Department of Clinical Neuroscience, Karolinska Institute, Stockholm, Sweden; 2 Capio Spine Center Stockholm, Löwenströmska Hospital, Upplands-Väsby, Sweden; 3 Clinical Science, Intervention and Technology (CLINTEC), Karolinska Institutet, Stockholm, Sweden; 4 Department of Surgical Sciences, Uppsala University, Uppsala, Sweden; 5 Department of Orthopedics and Hand Surgery, Uppsala University Hospital, Uppsala, Sweden; 6 Department of Medical Sciences, Örebro University, Örebro, Sweden

**Keywords:** Dysphagia, Health-related quality of life, Radiographic measurements, Dysphagia Short Questionnaire (DSQ), Occipitocervical fusion

## Abstract

**Purpose:**

The purpose of this study was to evaluate patient-reported outcome measures (PROMS) on dysphagia, health-related quality of life (HRQoL) and return to work after occipitocervical fixation (OCF). Postoperative radiographic measurements were evaluated to identify possible predictors of dysphagia.

**Methods:**

All individuals (≥ 18 years) who underwent an OCF at the study center or were registered in the Swedish spine registry (Swespine) between 2005 and 2019, and were still alive when the study was conducted, were eligible for inclusion. There was no overlap between the cohorts. Prospectively collected data on dysphagia (Dysphagia Short Questionnaire DSQ), HRQoL (EQ5D-3L) and return to work were used. Radiological and baseline patient data were retrospectively collected. In addition, HRQoL data of a matched sample of individuals was elicited from the Stockholm Public Health Survey 2006.

**Results:**

In total, 54 individuals were included. At long-term follow-up, 26 individuals (51%) had no dysphagia, and 25 (49%) reported some degree of dysphagia: 11 (22%) had mild dysphagia, and 14 (27%) had moderate to severe dysphagia. On a group level, the OCF sample scored significantly lower EQ_VAS_ and EQ-5D_index_ values compared to the general population (60.0 vs. 80.0, *p* = 0.016; 0.43 vs. 0.80, *p* < 0.001). Individuals working preoperatively returned to work after surgery. Of those responding, 88% stated that they would undergo the OCF operation if it was offered today. No predictors of dysphagia based on radiographic measurements were identified.

**Conclusion:**

Occipitocervical fixation results in a high frequency of long-term dysphagia. The HRQoL of OCF patients is significantly reduced compared to matched controls. However, most patients are satisfied with their surgery. No radiographic predictors of long-term dysphagia could be identified. Future prospective and systematic studies with larger samples and more objective outcome measures are needed to elucidate the causes of dysphagia in OCF.

**Supplementary Information:**

The online version contains supplementary material available at 10.1007/s00701-024-05991-6.

## Introduction

**Fig. 1:**
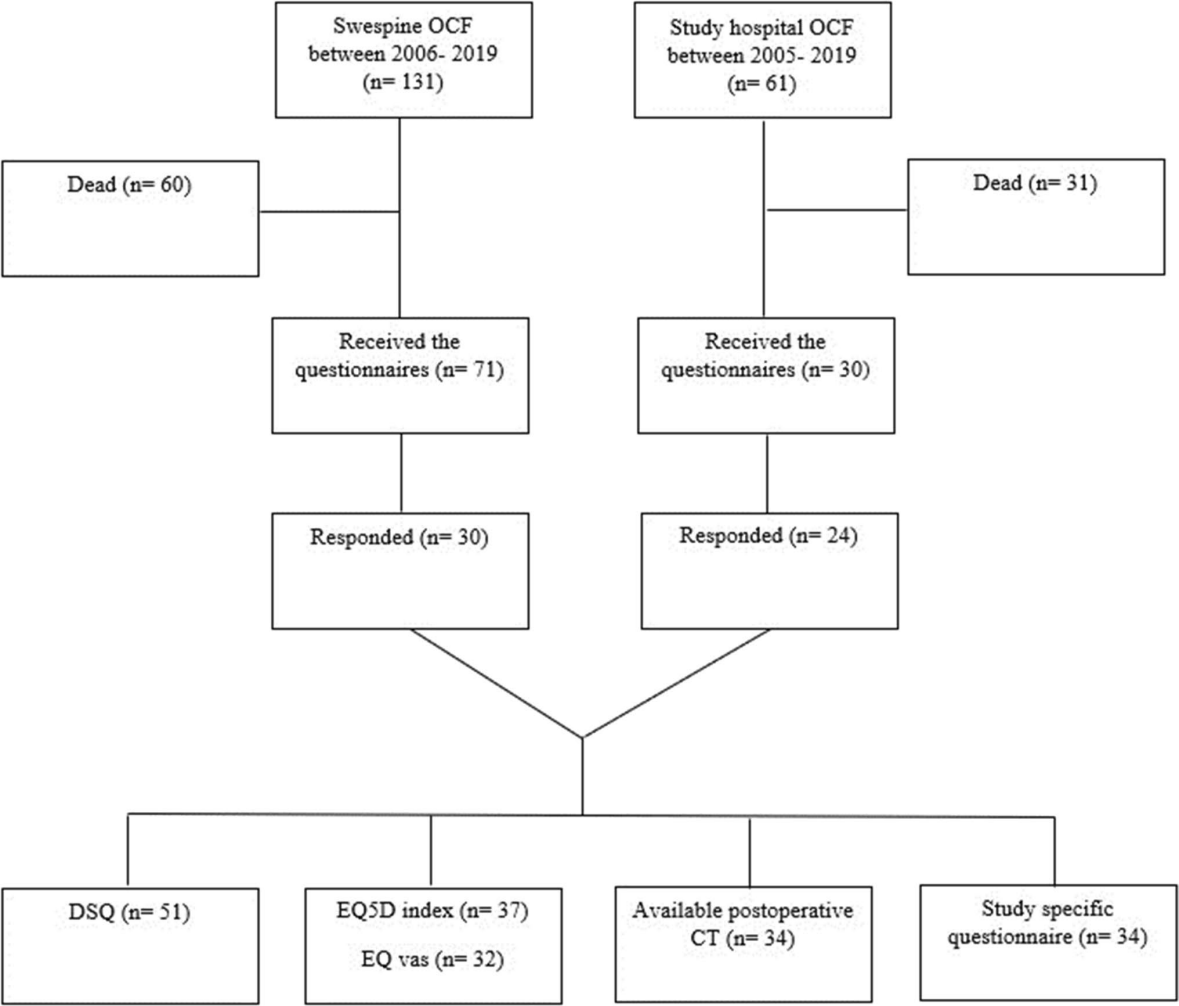
Flow chart illustrating the steps resulting in the inclusion of the final 54 cases and their questionnaire response frequencies

Occipitocervical fusion (OCF) is a treatment used to address various pathologies that cause instability in the craniocervical junction (CCJ) [17, 18, 21]. The CCJ is the most mobile segment of the spine and constitutes a complex anatomical region including the atlanto-occipital and the atlanto-axial joints [16, 17]. Surgical fusion of the CCJ greatly reduces mobility of the upper cervical spine. Furthermore, an OCF may extend beyond the CCJ, including lower cervical or even upper thoracic segments, further limiting spine mobility. Complications following the procedure may include non-union, spinal cord injury; CSF leaks; vertebral artery injury; wound infection; and dysphagia [5, 9, 31]. Dysphagia is a common complication and may be quantified using several scoring systems [1, 4]. Recently, it was shown that approximately 25% of patients undergoing OCF experienced dysphagia at short-term follow-up. However, at long-term follow-up (12–72 months), resolution of the swallowing difficulties was seen in one-third of these patients [28]. Efforts to predict and prevent postoperative dysphagia are crucial as this complication has been associated with prolonged hospital stay, reduced health-related quality of life (HRQoL) [25], and increased mortality [3, 11, 12, 20, 27, 30]. In a systematic review, several pre- and post-operative radiographic measurements were found to correlate with the occurrence of dysphagia in patients undergoing OCF [28] (Fig. 1). Although a C1-C2 fixation may in many cases be sufficient to achieve stability, this study did not aim to evaluate indications for OCF.

The aim of this study was to evaluate patient-reported outcome measures (PROMS) on dysphagia, health-related quality of life, and return to work after occipitocervical fixation. Postoperative radiographic measurements were evaluated to identify possible predictors of dysphagia.

## Materials and methods

### Study design, setting, and samples

This observational study used prospectively collected PROMS on dysphagia, HRQoL, and return to work. Radiological and baseline patient data were retrospectively collected. In addition, HRQoL data of a matched sample of individuals was elicited from the Stockholm Public Health Survey 2006.

### Hospital sample

The study hospital is a publicly funded and owned tertiary care center serving a region of roughly 2.3 million inhabitants. Individuals were identified using the surgical management software Orbit (Evry Healthcare Systems, Solna, Sweden). Medical records and imaging data from digital hospital charts were retrospectively reviewed using the health record software TakeCare (CompuGroup Medical Sweden AB, Farsta, Sweden) and regional electronic archives. All adult individuals (≥ 18 years) who underwent an OCF at the study center between 2005 and 2019 were eligible for inclusion. Sixty-one individuals were identified (Fig. 1). Thirty were still alive and were sent consent forms and study questionnaires by regular mail. The responding 24 (80%) individuals were included in the study.

### Swespine sample

Data from 2006 to 2019 collected by the Swedish Spine Registry (Swespine) was acquired. Swespine has a coverage of 98% (46 of 47 spinal clinics, the study center was the exception) [26]. In total, 131 individuals treated with OCF were identified in the registry. There was no overlap between this cohort and the hospital sample. Seventy-one individuals were still alive at the time of data collection for this study and were sent consent forms and study questionnaires by regular mail. Thirty (42%) responded and were included in the study (Fig. 1).

### General population sample for HRQoL comparisons

For matched HRQoL comparisons, a sample from the Stockholm Public Health Survey 2006 was used. This was a cross-sectional survey of the general population in Stockholm County. It was sent to 57,000 persons aged 18–84 years, had a response rate of 61%, and included a generic HRQoL questionnaire (EQ-5D-3L). Questions regarding the occurrence of long-term illness, disability, or other ailments were also part of the survey [2]. A 1:3 matching was used when comparing OCF and controls. Controls were matched for age and sex and randomly selected from the survey, without any restrictions for inclusion in the study. In all, 111 controls were identified and included.

### PROMS and outcome measures

#### The Dysphagia Short Questionnaire (DSQ)

The DSQ consists of five key domains which cover different aspects of dysphagia ([24], Supplementary Fig. 1). These are the ability to swallow, incorrect swallowing, lump feeling, involuntary loss of weight, and pneumonia, and each category has different grades of clinical severity. The score has been validated and was found to accurately reflect the severity of dysphagia. The total score is calculated by summing up the points in each category to a maximum of 18 points, where higher scores represent a greater severity of the symptoms [24]. We stratified the responses into no dysphagia (DSQ scores ≤ 2), mild dysphagia (DSQ > 2 but < 4.5), and moderate to severe dysphagia (DSQ scores ≥ 4.5), according to Liang et al. [15]. A total of 51 individuals answered the DSQ questionnaire.

#### EQ-5D-3L

The generic HRQoL instrument EQ-5D-3L consists of two parts. The first part is a descriptive system in which the respondents classify their health in five dimensions (mobility, self-care, usual activities, pain/discomfort, and anxiety/depression) within three severity levels (coded no problems = 1, moderate problems = 2, or severe problems = 3) [22]. The response value of each dimension generates a five-digit value representing a corresponding health state, which can be indexed into a single overall HRQoL value, EQ-5D-3L_index_. The EQ-5D-3L index runs from − 0.59 (worst) to 1.0 (best) [6, 14]. The second part is the EQ visual analogue scale (*EQ*_*VAS*_), where the respondents rate their current health between 0 (worst imaginable health) and 100 (best imaginable health). A total of 37 OCF individuals answered the EQ-5D-3L questionnaire.

#### Study-specific questionnaire

A set of study-specific questions was included and concerned employment status before and after OCF surgery and overall return to work. One question, concerning preference to undergo the same OCF operation again if it was offered, was also included. A total of 34 individuals answered this part of the questionnaire [2]. This study is reported in line with the STROBE Guidelines [29].

#### Radiographic measurements

**Fig. 2:**
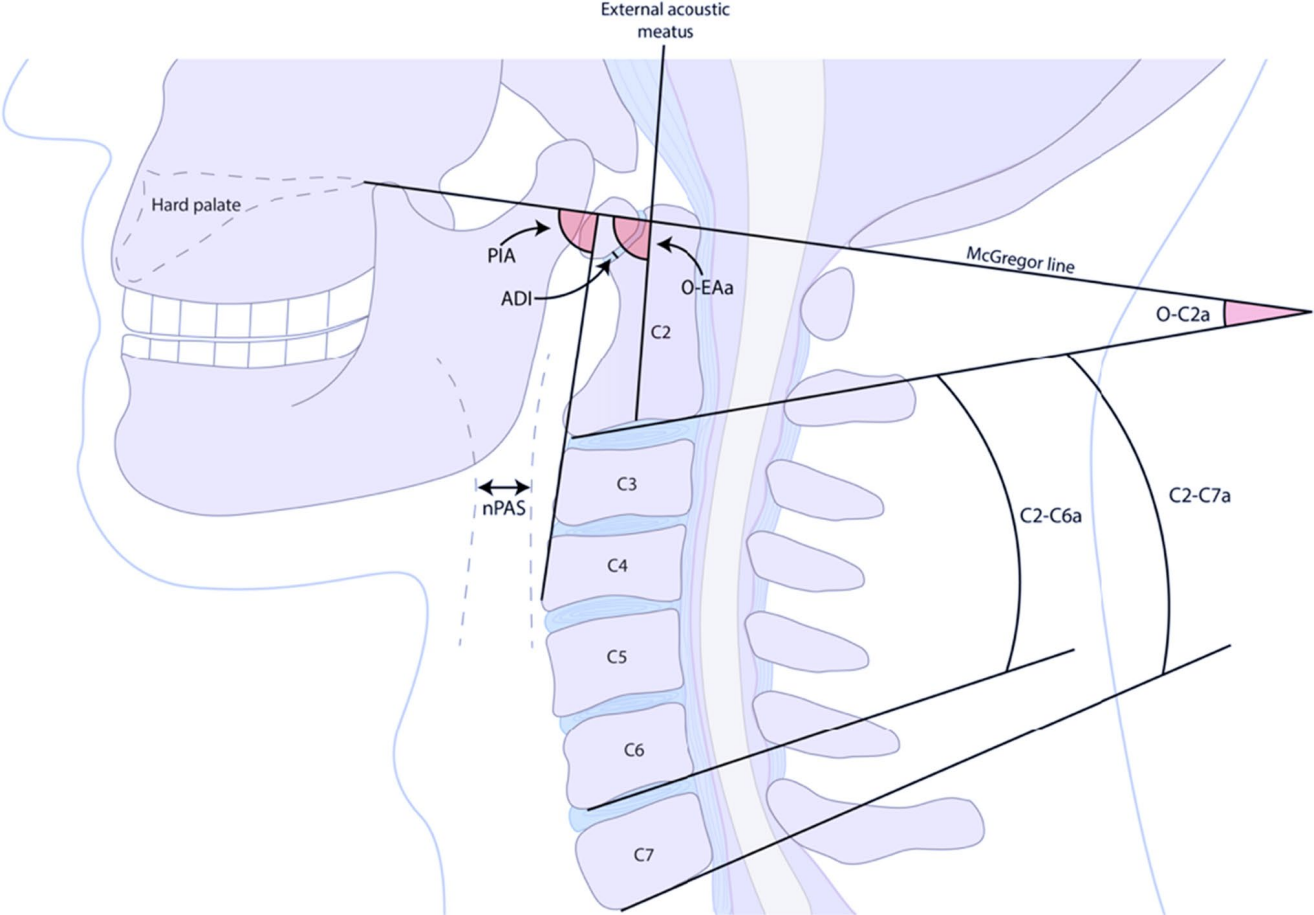
Radiographic measurements and angles commonly reported in relation to OCF and dysphagia. ADI, atlantodental interval; C2-C6a, C2-C6 angle; C2-C7a, C2-C7 angle; nPAS, narrowest oropharyngeal airway space; O-C2a, occipito-C2 angle; O-EAa, occipital and external acoustic meatus to axis angle; PIA, pharyngeal inlet angle

CT scans, when available, were requested from treatment centers. A total of 34 individuals had postoperative imaging available for radiographic measurements of distance and angles. All images were obtained on multidetector CT scanners (MDCT), either 64-MDCT (GE Lightspeed VCT, GE Healthcare, Waukesha, WI, USA and Philips Brilliance 64, Philips, Amsterdam, Netherlands) or 128-MDCT (Siemens Somatom Definition Flash, Siemens Healthcare, Forcheim, Germany). The protocols used a rotation time of 280–500 ms, tube voltage of 100–140 kV, and tube current of 40–97 mA. Multiplanar reformation of the acquired images was performed on a dedicated workstation using 3D imaging software (Sectra IDS7 23.1, Sectra, Linkoping, Sweden). Images were reconstructed into slabs of 1 mm, in three orthogonal planes: axial, coronal, and sagittal. To reconstruct the midsagittal plane, the crosshairs were aligned on the axial and coronal planes. Bone window settings were used on all images, with fixed Hounsfield units (HU) of a width of 2500 HU and a length of 250 HU. A radiologist performed all radiographic measurements (Fig. 2). The following eight measurements, recorded in distances (mm) or angles (degrees), were acquired in the midsagittal plane. (1) Atlantodental interval (ADI) is the distance between the posterior border of the anterior arch of the atlas and the anterior border of the dens. (2) Narrowest oropharyngeal airway space (nPAS) is the most constricted distance within the oropharynx, measured between the tips of the uvula and epiglottis. (3) Pharyngeal inlet angle (PIA) is the angle formed by the line connecting the midpoint of the anterior arch of C1 to the apex of the cervical sagittal curvature and McGregor’s line between the posterior border of the hard palate and the most inferior point of the occipital curve. (4) Occipital and external acoustic meatus-to-axis angle (O-EAa) is the angle between McGregor’s line and the line connecting the midpoints of the external acoustic meatus and the inferior endplate of C2. (5) Occipito-C2 angle (O-C2a) is the angle between the McGregor’s line and the midpoint of the inferior endplate of C2. (6) C2 tilting angle (C2Ta) is the angle between the inferior endplate of C2 and a line that joins the midpoint of the external acoustic meatus with the midpoint of the inferior endplate of C2. (7) C2-C6 angle (C2-C6a) is the angle between the inferior endplate lines of C2 and C6. (8) C2-C7 angle (C2-C7a) is the angle between the inferior endplate lines of C2 and C7.

### Statistical analysis

Continuous data were presented as mean (standard deviation), non-parametric data as median (interquartile range, IQR), or numbers (percentage). The Shapiro–Wilk test was used to evaluate the normality of continuous data. The Mann–Whitney *U* and Chi-square or Fisher’s exact tests were used to compare continuous and categorical variables between groups as appropriate. Correlation matrices as well as the Spearman correlation coefficient were used to investigate the association between radiographic angles and DSQ. Uni- and multivariable logistic regression was used to identify predictors of dysphagia. EQ-5D-3L data were compared between the OCF sample and the general population sample, and sub-group comparisons were made between males and females. To analyze differences between groups, Fisher’s exact test and the independent samples median test were used. Moderate and severe levels on EQ-5D-3L dimensions were collapsed before analysis. When analyzing employment status and return to work, comparisons were made between individuals having mild dysphagia (defined as DSQ > 2) and those having moderate to severe dysphagia (DSQ ≥ 4.5). All analyses were conducted using the statistical software programs R and SPSS. Statistical significance was set at *p* < 0.05.

## Results

### Baseline data

A total of 54 individuals were included in this study after undergoing an OCF, with a median age of 62 years. The median BMI was 23.6 (IQR, 22–26), and the median number of instrumented levels for the OCF was 4.0 (IQR, 3.0–7.0). The median follow-up was 9.2 years (6.9–12). The most common diagnoses leading to OCF were rheumatoid arthritis (39%), followed by fractures (22%) and cervicalgia (13%). No congenital anomalies or platybasia were found. Only two patients had undergone prior anterior surgery; however, none of them had any dysphagia prior to their OCF surgery. These were a case of chordoma treated with anteroposterior surgery with a DSQ of 3 and a case of anterior cervical discectomy and fusion C5-C7 25 years before OCF, with a DSQ of 9. Dysphagia was not the indication for surgery, and none of the patients had dysphagia prior to surgery.

**Fig. 3:**
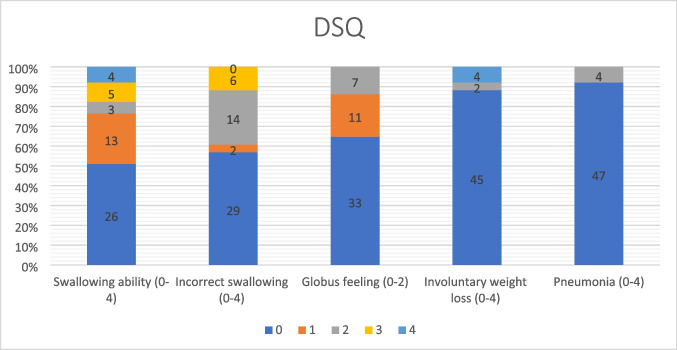
Bar chart showing the frequency of the scores on each of the five DSQ score domains. Each response category represents different grades of clinical severity, where 0 is no problems and 4 severe problems (supplementary Table 1)

The median total DSQ score was 2.0, and a total of 51 individuals answered the DSQ questionnaire. Based on the DSQ scores at long-term follow-up, 26 individuals (51%) had no dysphagia, and 25 (49%) reported some degree of dysphagia: 11 (22%) had mild dysphagia, and 14 (27%) had moderate to severe dysphagia (Fig. 3).

### EQ-5D-3L index

The OCF sample exhibited significantly poorer outcomes compared to the general population sample in the three dimensions, mobility, self-care, and usual activities (Table 1). On a group level, the OCF sample scored significantly lower *EQ*_*VAS*_ values compared to the general population (60.0 vs. 80.0, *p* = 0.016). The OCF sample showed significantly lower *EQ-5D*_*index*_ values (0.43 vs. 0.80, *p* < 0.001), and six individuals (16%) reported a negative index value, worse than death, compared to none in the general population sample. Six individuals (16%) in the OCF sample reported an index value of 1.00, indicating perfect health, compared to 37 individuals (33%) in the general population sample.

**Table 1:** Percentage (number) of respondents reporting no, moderate, or severe problems in EQ-5D dimensions, *EQ-5D*_*index*_ and *EQ*_*VAS*_, OCF sample, and general population sample

EQ-5D dimensions	Overall population		
	OCF (*n* = 37)	General Population (*n* = 111)	*p value*
Mobility			< 0.001
No problems	12 (32%)	84 (76%)	
Moderate problems	20 (54%)	27 (24%)	
Severe problems	5 (14%)	0 (0%)	
Self-care			< 0.001
No problems	21 (57%)	104 (94%)	
Moderate problems	6 (16%)	6 (5%)	
Severe problems	10 (27%)	1 (1%)	
Usual activities			< 0.001
No problems	11 (30%)	91 (82%)	
Moderate problems	12 (32%)	19 (17%)	
Severe problems	14 (38%)	1 (1%)	
Pain/discomfort			0.108
No problems	8 (22%)	41 (37%)	
Moderate problems	19 (51%)	68 (61%)	
Severe problems	10 (27%)	2 (2%)	
Anxiety/depression			0.207
No problems	24 (65%)	84 (75%)	
Moderate problems	11 (30%)	24 (22%)	
Severe problems	2 (5%)	3 (3%)	
Median *EQ-5D*_*index*_ (IQR)	0.43 (0.07–0.75)	0.80 (0.73–1.0)	< 0.001
Median *EQ*_*VAS*_ (IQR)	60.0 (38.8–80.0)^c^	80.0 (60.0–90.0)	0.016

### Employment status and return to work after an OCF

A total of 34 OCF individuals responded to the study-specific questionnaire concerning employment status. The employment status for 25 (78%) individuals was either retired or on sick leave preoperatively. Only seven (22%) individuals were working either full time or part time (50–75%), and all of them returned to work after surgery. Among the 34, 30 (88%) individuals stated that they would undergo the OCF operation if it was offered today. The remaining four individuals that stated that they would not undergo OCF again reported that they had experienced complications or no improvement after surgery.

### Predictors of dysphagia

There was no significant difference in the baseline data or postoperative radiographic angles between individuals with and without dysphagia (Tables 2 and 3). There was no significant correlation between DSQ results and postoperative radiographic measurements. No predictors of dysphagia based on radiographic measurements were identified.

**Table 2:** Univariable logistic regression predicting mild dysphagia

Characteristic	Number	OR	95% CI	*p* value
Age	51	1.00	0.96–1.05	0.986
Number of instrumented levels	48	1.14	0.94–1.41	0.202
Height (cm)	42	0.95	0.89–1.00	0.090
Weight (kg)	42	0.97	0.93–1.01	0.201
BMI	42	0.97	0.83–1.13	0.701
ADI (mm)	32	0.88	0.43–1.69	0.697
nPAS (mm)	32	1.07	0.87–1.33	0.515
PIA	31	1.01	0.94–1.09	0.714
O-EAa	32	1.02	0.92–1.13	0.728
C2Ta	32	1.01	0.94–1.10	0.747
O-C2a	32	0.99	0.91–1.07	0.804
C2-C6a	26	1.00	0.93–1.08	0.998
C2-C7	22	1.01	0.94–1.10	0.798

**Table 3:** Univariable logistic regression predicting moderate/severe dysphagia

Characteristic	Number	OR	95% CI	*p* value
Age	51	1.01	0.96–1.06	0.827
Number of instrumented levels	48	1.04	0.83–1.26	0.736
Height (cm)	42	0.95	0.88–1.01	0.114
Weight (kg)	42	0.98	0.93–1.02	0.353
BMI	42	1.01	0.85–1.20	0.892
ADI (mm)	32	0.71	0.26–1.51	0.430
nPAS (mm)	32	1.26	1.00–1.70	0.076
PIA	31	1.02	0.94–1.12	0.590
O-EAa	32	1.08	0.97–1.22	0.163
C2Ta	32	1.07	0.98–1.18	0.122
O-C2a	32	1.00	0.91–1.09	0.926

## Discussion

In this study on outcomes after OCF surgery, 27% reported a moderate to severe dysphagia at a median of 9 years of follow-up. The OCF cases reported significantly poorer HRQoL compared to the general population sample, in three out of five EQ5D dimensions. The few individuals working full or part time before surgery returned to work after the procedure. The patients did not have preexisting dysphagia. There were no cases with malformations or congenital anomalies. The pharyngooesophageal angulation was studied using radiographic measurements. No predictors of dysphagia were identified based on radiographic measurements.

Previous studies have reported 10–25% dysphagia after OCF at long-term follow-up, and our results are in line with these reports [28]. In this study, 53% of the individuals reported any dysphagia and 27% moderate to severe dysphagia. Our slightly higher dysphagia numbers may be explained by differences between dysphagia assessment tools. DSQ is an excellent tool to detect mild dysphagia symptoms with high sensitivity and specificity [15]. However, most previous studies have used the Bazaz scale, which has a lower sensitivity and may underdiagnose the condition [15].

Pathologies with instability in the craniocervical junction can cause neck pain, functional limitations, and reduced HRQoL [19]. To the best of our knowledge, there are no studies addressing HRQoL after OCF surgery. One meta-analysis has evaluated the HRQoL of individuals undergoing either anterior or posterior decompression for degenerative cervical myelopathy [23]. Their conclusion was that both posterior and anterior decompressions were similar in terms of functional outcome after a follow-up period of 1 year [23]. However, none of the patients in the posterior decompression group had undergone OCF. The findings highlight the need for studies investigating the long-term follow up and HRQoL after OCF surgery.

In this study, the OCF sample scored significantly poorer HRQoL in comparison to the general population sample, even though respondents randomized from the general population sample may suffer long-standing illness or disabilities. The OCF sample scored significantly more problems within the dimensions mobility, self-care, and usual activities, which resulted in significantly lower EQ-5D_index_. Moderate problems in the mobility dimension mean having some problems to walk, and severe problems mean being confined to bed. Scoring moderate problems in the self-care dimension corresponds to having some problems washing or dressing, while severe problems mean being unable to do so. The usual activities dimension refers to the ability to work, study, and participate in family or leisure activities [8]. Thus, we can conclude that the OCF individuals’ overall mobility was affected and impacted the ability to walk, manage hygiene, and participate in daily activities. In addition, a large proportion of them suffered from dysphagia, which in qualitative interviews has been described to negatively affect quality of life, especially during meals [25].

Regarding return to work, the OCF sample size was too small to allow for any valid conclusions. Among the 34 individuals that responded, a majority was either retired or on sick leave before the surgery. Of note, the median age of the whole group was 62 years. Only seven individuals were working preoperatively, all of them returned to work within 6 months after the surgery. Interestingly, 88% of the individuals were satisfied with their surgeries and would accept to undergo the OCF procedure if it was offered today.

Based on the findings of this study, there was not enough evidence to establish a correlation between dysphagia (DSQ) and any of the radiographic measurements studied. This may be explained by any of the following: (1) DSQ is a poor estimate of dysphagia, (2) all individuals in this sample were fused in acceptable angles, (3) the measured angles are not relevant to dysphagia. While the first alternative is unlikely as the DSQ has been validated repeatedly [10, 24], the other alternatives merit further consideration.

As reported in a recent review [28], it is unclear whether radiographic measurements can predict dysphagia after OCF. However, the same review highlighted a few radiographic angles, including postoperative PIA, postoperative O-EAa, and postoperative nPAS, as plausible predictors of post-OCF dysphagia (Fig. 2) [28]. In this study, these measurements were evaluated in relation to DSQ but failed to find any correlation.

Dysphagia after OCF is most likely multifactorial. Proposed mechanisms include pharyngeal edema in the immediate postoperative phase, lack of neuromuscular coordination of swallowing muscles or narrowing of oropharyngeal space in extreme flexion or extension of the cervical spine, and other biomechanical factors [7, 11, 13, 18].

Although diagnosis and treatment of dysphagia were outside the scope of this manuscript, we suggest that patients with dysphagia secondary to neurosurgical interventions should be examined by Logopedics and ENT consultants and undergo repeated examinations. The treatment should be designed individually and tailored based on the identified causes.

### Limitations

This study has several limitations. The most important one concerns missing data. The study collected data from three different sources where the available variables differed. The included sample size is still small, despite the pooling of local hospital data and nation-wide registry data. The overall frequency of responders is low, 80% in the institutional data and 44% from the Swespine registry. Furthermore, there are limitations inherent to patient-reported data originating from voluntary surveys; there may be a selection-bias or attrition-bias between those who respond to questionnaires and those who opt out. Nonetheless, this is the third largest cohort of OCF patients reported in the literature and the only one using DSQ and including HRQoL and return to work data.

There is a need for prospective, multicenter studies, addressing the issue of dysphagia and OCF in a systematic manner. We propose a study protocol including pre- and postoperative standing radiographs and combining dysphagia questionnaires with fluoroscopic swallowing examinations and functional endoscopic evaluation of swallowing (FEES). Pre- and postoperative assessment of HRQoL, including long-term follow-up, should also be considered.

## Conclusion

Occipitocervical fixation results in a high frequency of long-term dysphagia. The HRQoL of OCF patients is significantly reduced compared to matched controls. However, most patients are satisfied with their surgery. No radiographic predictors of long-term dysphagia could be identified. Future prospective and systematic studies with larger samples and more objective outcome measures are needed to elucidate the causes of dysphagia in OCF.

## Supplementary Information

Below is the link to the electronic supplementary material.

Supplementary file1 (DOCX 16 KB)

## Data Availability

The datasets generated during the current study are available from the corresponding author on reasonable request.

## Code availability

No specific software codes used.
