# Supplementary material for: Dysphagia, health-related quality of life, and return to work after occipitocervical fixation
Source: Acta Neurochir (Wien). 2024 Feb 20;166(1):90. doi: 10.1007/s00701-024-05991-6 (PMC10876741; doi:10.1007/s00701-024-05991-6)
Supplement: Supplementary file 1 — Supplementary file1 (DOCX 16 KB) [file 701_2024_5991_MOESM1_ESM.docx]

**Supplementary table 1:** The DSQ questionnaire provided by Dr. Martin Skeppholm [24].

## Appendix

## The Dysphagia Short Questionnaire

Please tick one box only, under each heading

| **Ability to swallow** | Points |
| --- | --- |
| I have no difficulty in swallowing | 0 |
| Food occasionally gets stuck in my throat if I have not chewed it thoroughly enough | 1 |
| I find it hard to swallow solid food | 2 |
| I find it hard to swallow liquid food | 3 |
| I find it hard to swallow saliva | 4 |
| **Incorrect swallowing** |  |
| I do not feel that I swallow incorrectly | 0 |
| I sometimes feel I’m swallowing incorrectly, though it does not cause me to cough | 1 |
| I sometimes cough in connection with swallowing | 2 |
| I frequently cough in connection with swallowing | 3 |
| I always get a fit of coughing when I swallow | 4 |
| **Lump feeling** |  |
| I do not have the feeling there is a lump in my throat | 0 |
| I sometimes have the feeling there is a lump in my throat | 1 |
| I always have the feeling there is a lump in my throat | 2 |
| **Involuntary loss of weight** |  |
| I have not lost weight recently | 0 |
| I have lost three or four pounds recently | 2 |
| I have lost more than five pounds recently | 4 |
| **Pneumonia** |  |
| I have not had pneumonia | 0 |
| I have had the occasional bout of pneumonia | 2 |
| I have had repeated bouts of pneumonia | 4 |
